# What Science Has to Settle

**Published:** 1868-02

**Authors:** 


### WHAT SCIENCE HAS TO SETTLE.

The unity of the human race has ever been a stumbling block to science, notwithstanding the subject has been under review for centuries, and is likely to remain so, for aught we can see to the contrary, for centnries to come, or until we have a new revelation, so convincing that wise and foolish will be forced to a common belief.

As a social question it has little intrinsic merit. The status of the negro is fixed. His unmoveable character stamps him a barbarian of barbarians. Occupying a country of vast extent and resources, he has no system of labor, no commerce, has built no cities, has produced no statesmen or philosophers, established no schools, has no language either written or spoken, and in short he is in his own country to-day what he was thirty centuries ago, a savage.

His introduction into America, brought about as it was by avarice and held as a slave for centuries, for no higher motive, until slavery became an institution which humanity as well as interest shrank from destroying, has nevertheless furnished an insight into his character, which but for it could not have been known, and shows convincingly his adaptibility to certain great ends of existence, and no less his non-adaptibility to others. He is to-day, more than ever, a study for the wise and curious, and should be an object of sympathy with the good. In his subordinate position he is mild and imitative; shows ability to master the lower branches of fractional mathematics, agriculture and domestic labor, but with little or none to acquire land or accumulate an estate. He loves holidays and shows, jewelry and feathers, sings, whistles and dances from intuition, has a degree of honesty and integrity, and may be trusted to a certain extent, but no further. As a ruler he is unreasoning and cruel. The Drivers, as the negro overseers were called on the plantations, were the severest task masters, and generally punished with the greatest rigor. They learn rapidly so far as they are able to imitate, but beyond imitation they show little or no capacity for progress.

The mixed bloods possess more sprightliness, but fewer virtues and less endurance. As the Indian never ceases to be an Indian; so the negro never ceases to be a negro. But whilst this is true, the question remains unanswered, who is he?—what is his origin?—what his physical status, and what is to be his destiny?

The church universal we believe, stakes itselef on the affirmation of the unity of man. Whilst many of the greatest naturalists, physiologists and pathologists take the other side and assert that he is not only not of the Adamic race, but a beast, and without a soul.

God has given us two books—Nature and Providence—these the more closely they are examined the more closely are they found to harmonize. If there is a discrepency it is not from any fault in the books, it is in man. We see the sun, the moon, and the stars, all beyond this we call immensity, simply because we know nothing about it. We frequently discredit the laws of nature and the teachings of revelation for the same reason. History, the sure foundation of philosophy, is discredited to make an absurdity plausible. That man is an enemy to his race who permits history, the teachings of revelation, or the laws of nature, or stops short of truth, that he may become a leader of thought for a time.

It is within the scope of Science to settle this question. Revelation, history, philosophy, geology, minerology, chemistry, electricity, anatomy, physiology, and pathology must all play a part in the grand illumination that will take place when the truths now being discovered in each of these departments shall flood the world with light.

The true idea of distinction among the types of man never having been reached, all opinions on this subject are purely provisional, and liable to be swept away at any moment by the discovery of new facts changing in toto the extreme opinions on both sides, which have of late been industriously promulgated by overzealous advocates.

For forty centuries the negro has remained the same—his distictive marks and phenomena of existence are unaltered, and unalterable; a great question has yet to be decided by him and through him. The time may be postponed, but it will nevertheless come, when the beginning and the end of the negro will be indisputably revealed, and history and science vindicated.

Then let us wait and work; truths are accumulating and earnest men are at work unfolding the laws of nature which will in turn clear up the mystery that envelopes the subject and vindicate the ways of God to man.

